# Adherence to Antihypertensive Medications in Iranian Patients

**DOI:** 10.1155/2016/1508752

**Published:** 2016-03-16

**Authors:** Azin Behnood-Rod, Omid Rabbanifar, Pirouz Pourzargar, Alireza Rai, Zahra Saadat, Habibollah Saadat, Yashar Moharamzad, Donald E. Morisky

**Affiliations:** ^1^Islamic Azad University, Tehran Medical Branch, Tehran, Iran; ^2^Zanjan University of Medical Sciences, Zanjan, Iran; ^3^Cardiovascular Research Center, Shahid Beheshti University of Medical Sciences, Tehran, Iran; ^4^School of Medicine, Kermanshah University of Medical Sciences, Kermanshah, Iran; ^5^Department of Community Health Sciences, UCLA Fielding School of Public Health, Los Angeles, CA, USA

## Abstract

*Introduction*. Appropriate adherence to medication is still a challenging issue for hypertensive patients. We determined adherence to antihypertensive(s) and its associated factors among 280 Iranian patients.* Methods*. They were recruited consecutively from private and university health centers and pharmacies in four cities. The validated Persian version of the 8-item Morisky Medication Adherence Scale (MMAS-8) was administered to measure adherence.* Results*. Mean (±SD) overall MMAS-8 score was 5.75 (±1.88). About half of the sample (139 cases, 49.6%) showed low adherence (MMAS-8 score < 6). There was a negative linear association between the MMAS-8 score and systolic BP (*r* = −0.231, *P* < 0.001) as well as diastolic BP (*r* = −0.280, *P* < 0.001). In linear regression model, overweight/obesity (*B* = −0.52, *P* = 0.02), previous history of admission to emergency services due to hypertensive crisis (*B* = −0.79, *P* = 0.001), and getting medication directly from drugstore without refill prescription in hand (*B* = −0.51, *P* = 0.04) were factors recognized to have statistically significant association with the MMAS-8 score.* Conclusion*. Antihypertensive adherence was unsatisfactory. We suggest that health care providers pay special attention and make use of the aforementioned findings in their routine visits of hypertensive patients to recognize those who are vulnerable to poor adherence.

## 1. Introduction

Hypertension (HTN) is a major public health challenge in many parts of the world [1]. Left uncontrolled, HTN poses serious health problems on sufferers including heart attack, heart failure, and stroke [2]. Health care providers should make use of all armamentariums available for better control of HTN. Beside regular checkups, medication intensification, health surveillance, and patient education, a factor that health care providers should be more aware about in facing this population is assessing the extent to which hypertensive patients comply with medications as prescribed for them (i.e., adherence to medication) [3].

Considering the fact that there are effective medications available to control HTN and the aggressive stance required with hypertensive patients, we think that assessing the issue of medication adherence could be an integral part of management of such patients. In other words, medication adherence, although a complex issue with various associated factors, is a very critical component in controlling blood pressure (BP) of patients within the recommended normal ranges and consequently achieving good health outcome in long term and holding up the cardiovascular complications [4]. Nonadherence has been noted as one of the contributing factors in uncontrolled BP [5].

The pertinent literature review from different countries revealed that adherence to medications prescribed for patients with high BP may not be satisfactory. Good/acceptable adherence, determined via various tools, has been reported as 15.8% [6], 20.3% [7], 43.1% [8], 43.7% [9], 46% [10], 53% [11], 58% [12], and 77% [13]. As seen, most studies reported that good adherence to antihypertensives was documented in half of the patients studied. Most of the aforementioned studies also conceded that poor medication adherence was significantly associated with uncontrolled BP.

Hitherto, studies regarding medication adherence among hypertensive patients from Iran are insufficient. There are reports that a substantial proportion of patients have uncontrolled BP, reportedly 56.3% [14], 62% [15], and 65% [16]. In a comparative analysis carried out on data from national surveys of 20 countries, Iran was recognized as one of the countries with poor diagnosis and control of HTN [17]. With respect to lack of enough information about this issue in Iran, we intended to determine the antihypertensive adherence status of a sample of Iranian patients with HTN and find out any allied significant factor contributing to low medication adherence.

## 2. Materials and Methods

This cross-sectional study lasted from August to December 2014. This was a multicenter study including tertiary referral center of university-affiliated hospital, private cardiology office, pharmacy, and private general practitioner office in the cities of Tehran, Karaj, Kermanshah, and Bafgh. Inclusion criteria were adult patients of either gender who had documented HTN and were taking antihypertensive medications. The patients were interviewed directly at health centers or offices upon their presentation for checking their BP or to refill their prescriptions. A coordination session was held before the study initiation and the participating doctors and pharmacists were instructed by the principal investigator to interview any patient meeting the inclusion criteria consecutively during their daily visit of the patients in predetermined research locations. The pharmacists were asked to refer the eligible subjects to one of the nearest physicians involved in this research. For known patients for whom a medical record existed, in addition to the interview, other pertinent data were extrapolated from their records and were documented in the forms.

To determine the adherence to antihypertensives, the Persian version of the MMAS-8 which was validated by our team in a previous study was administered to the patients. The MMAS-8 was developed as a simple and reliable tool which can be used by clinicians to determine the adherence of patients to prescribed medications [18]. The 8-item scale was originally studied in hypertensive patients and the results revealed that it was a reliable (*α* = 0.83) tool and showed significant correlation with BP control (*P* < 0.05). It showed a sensitivity of 93% in detecting patients with poor BP control [19]. In our previous study on 200 Iranian patients who were suffering from HTN, we determined the Persian version of the MMAS-8 to be valid and reliable tool with an overall Cronbach's *α* coefficient of 0.697 and test-retest reliability showed good reproducibility (*r* = 0.940) [20].

In addition to the MMAS-8, a checklist was designed by the authors to gather demographic as well as variables about other diseases or medications the patients were taking. First, the MMAS was completed by the patients. If the patient was illiterate, the researcher read the questions for the patient and asked him/her to answer the questions orally. Then the correspondent answer was marked by the researcher on the MMAS and checklist. After completing the MMAS, the checklist data was completed. The data included in the checklist were demographic data (age, gender, weight, height, occupation, and educational level), duration of HTN, medications prescribed for HTN, other comorbidities, other medications other than antihypertensives, and control of HTN during the last 6 months by a health care provider. One of the variables included in the checklist was history of HTN crisis, in the form of emergency or urgency, leading to emergency admission. This variable was investigated using the medical records available at the research location or those from other medical centers in the past. Only documented reports/discharge notes signed by medical professionals containing detailed information were used to decide the previous history of HTN crisis. At the end, BP of the patients was measured by the researchers using a sphygmomanometer on the left arm when the patient was in seated position. The patients were asked to seat relaxed and not smoke for half an hour before BP recording. Korotkoff sounds were the basis to define systolic and diastolic BP. For the purpose of this study, brand new sphygmomanometers were provided to the doctors and an educational video was displayed and reviewed to ensure the lowest level of variation between BP readings by the research team members.

### 2.1. Statistical Analyses

All gathered data were entered into the SPSS software for Windows (ver. 18.0) (SPSS Inc., Chicago, IL). To express the results, we used descriptive indices including mean and its standard deviation (SD), frequency and percentage. Regarding the MMAS-8 score, we divided the patients into two groups of low adherence (i.e., MMAS-8 score of less than 6) versus moderate/high adherence (i.e., MMAS-8 scores of 6–8). The Chi-squared and Mann-Whitney *U* tests were applied to compare, respectively, the gathered categorical and continuous data between these two groups of MMAS-8 scores. To find any relationship between controlled BP (i.e., systolic BP < 140 and diastolic BP < 90 mmHg in nondiabetics and < 130/80 mmHg in diabetics) and MMAS score, the Pearson correlation test was used. Significance level was set at 0.05. To find correlation between systolic and diastolic BP values and the MMAS-8 score, Spearman's rho test was used. A multiple logistic regression analysis was done to predict effective variables that can affect poor drug adherence. In this analysis, all variables were indicated as independent and poor drug adherence (i.e., MMAS score < 6) was indicated as the dependent variable.

### 2.2. Ethics

Firstly, the objective of the study was explained to the patients. If the patient was interested in contributing to the study and gave his/her oral consent, then instructions about how to complete the MMAS-8 and the demographic form were provided to them. They were assured that the information will be used only for scientific purposes. The protocol of the study was in conformity with the Helsinki Declaration.

## 3. Results

In total, 280 patients were included. Mean (±SD) age of the participants was 60.3 (±10.0) years, 118 cases (42.1%) were male, 204 (72.9%) were overweight/obese (BMI values of ≥25 kg/m^2^), 164 (58.5%) had education level below high school diploma or were illiterate, and 49 (17.5%) were current smokers. Mean (±SD) overall MMAS-8 score was 5.75 (±1.88). About half of the sample (139 cases, 49.6%) showed low adherence to antihypertensives. Ninety-five cases (33.9%) had moderate adherence and 46 cases (16.4%) showed high adherence.

Table 1 presents demographic characteristics of the sample and comparison of these data between low and medium/high adherence groups. As depicted, there was higher percentage of patients with BMI values of less than 25 kg/m^2^ in moderate/high adherence group compared to low adherence group. Also, those who had received academic training and had occupations as clerk or in military personnel represented better adherence. However, considering other demographic information including gender, age, having an insurance coverage, residential place (urban areas versus rural ones), and being a smoker did not reveal significant difference between low and moderate/high adherers.

Mean (±SD) duration of HTN was 7.2 (±5.9) years (range, 6 months to 40 years). General practitioner (67 cases, 23.9%) and cardiologist (65 cases, 23.2%) were the most common presented physicians whose patients reported that they visit to control their high BP. Others reported visiting internist (24 cases, 8.6%), nephrologist (9 cases, 3.2%), or more than one physician to check their BP (115 patients, 41.0%). According to the criteria, 122 patients out of 280 cases (43.6%) had controlled BP. Among diabetics (i.e., 55 hypertensive diabetics with/without other concomitant conditions), only 2 cases (3.6%) had controlled BP.  Table 2 shows comparison of BP-related variables between low and moderate/high adherers. As seen, those who were low adherers had higher percentage of hypertensive crisis (hypertensive emergency or urgency) which required admission to hospital emergency services, lower rate of visiting their doctors to get their BP checked during the preceding 6 months from the study, and higher systolic and diastolic BP measurements when compared to moderate/high adherers. Controlled BP was also significantly higher in moderate/high nondiabetic adherers compared to those who had low MMAS-8 score. About diabetics, since the sample size was not enough, statistical significance was not seen.

Nearly half of the cases (127 patients, 45.3%) were taking one class of antihypertensive for their condition. Among these, angiotensin-receptor blocker (74 cases, 26.4%) was the most prevalent medication used, followed by selective beta-blockers (22 cases, 7.9%), hydrochlorothiazide (13 cases, 4.6%), angiotensin-converting enzyme inhibitor (10 cases, 3.6%), calcium-channel blocker (7 patients, 2.5%), and finally alpha-blocker (one patient, 0.4%). Others (153 cases, 54.6%) were taking more than one class of antihypertensive to control their high BP. One-hundred sixty patients (57.1%) had a concomitant condition other than HTN. The most common comorbidity was ischemic heart disease (with or without having undergone coronary artery bypass grafting (CABG) or minimally invasive percutaneous interventions; PCI) which was documented in 28 patients (10.0%). Then, diabetes mellitus (23 cases, 8.2%) and dyslipidemia (12 cases, 4.3%) were, respectively, most common comorbidities. Most patients (212 patients, 75.7%) reported that they had presented to pharmacies during the last 6 months to take their medication without controlling their BP by their doctor. In Table 3, comparison of antihypertensives taken and comorbidities between low and moderate/high adherers is presented. As observed, no association was detected between number of antihypertensives or number of comorbidities and medication adherence categories. The only statistically significant association was observed regarding those who reported that they personally without visiting their doctors requested the pharmacist to refill their prescription or in some cases, even without prescription in hand, purchased their medication directly from the drugstore.

There was a negative linear association between the MMAS-8 score and systolic BP (*r* = −0.231, *P* < 0.001) as well as diastolic BP (*r* = −0.280, *P* < 0.001).  Table 4 shows linear regression model results. Being overweight or obese, previous history of admission to emergency services due to hypertensive crisis, and getting medication directly from drugstore without refill prescription in hand were factors recognized to have statistically significant association with the MMAS-8 score.

## 4. Discussion

Good adherence to antihypertensives is a key factor in achieving appropriate BP control in hypertensive patients. The results obtained here show that compliance with antihypertensive(s) is not satisfactory among Iranian hypertensive patients. The rate of controlled BP and the significant association detected between systolic and diastolic BP measurements with adherence level support this finding. In other words, poor adherence affected negatively BP control. Adherence studies from Iran are not sufficient in terms of quantity and quality. The limited literature has noted diverse designs and tools to define adherence and subsequently direct comparison with the current results may not be feasible. According to a review article, though there has been good effort to determine adherence to diabetes medicines, no high quality study has been done so far in Iran on hypertensive patients. In particular, the tools used to define adherence included pill counting and self-reported questionnaire [21]. It has been noted that methods such as 24-hour recall and refill history do not accurately measure medication adherence [22]. We administered the MMAS-8 to define medication adherence. This scale has been established in several studies across different cultures and languages and various populations to be a reliable tool in determining adherence [6, 7, 23–25]. As mentioned above, we observed a good reliability and validity of this scale in Persian speaking patients in a previous study [20].

In a former study on 250 patients with high BP in Shiraz city, Iran, using a self-report questionnaire and ratio of taken pills to prescribed pills in one-month period, it was found that having a positive attitude toward antihypertensives and the interval between visits to physician of less than 3 months were two independent predictors of good adherence [26]. We did not study attitude or knowledge of the patients here, but about 40% of the patients reported that they had visited more than one doctor to control their BP. Adding this to the figure of one-fifth of the cases who had never scheduled an appointment to visit their doctors during the preceding 6 months of the study may reflect faults in health care system. Reviewing the literature, it seems that there is no general consensus among experts and scientific organizations about visit frequency in hypertensive patients, but studies confirm the fact that shorter intervals yield better BP control and earlier normalization [27, 28].

In a study on older adults with HTN and using the Morisky 4-Item Self-Report Measure of Medication-Taking Behavior (MMAS-4), the authors found that older people with a comorbidity and longer history of HTN had better adherence. These are in contrast to our finding as age, presence of comorbidity or its number and duration of HTN were not different between poor and moderate/good adherers. This may be due to different scales to define adherence (MMAS-4 in Chinese study versus MMAS-8 in our study) and the nature of patients studied in the mentioned article which included only those who aged more than 55 years. The only similar finding was higher educational level in both studies which was associated with better adherence. The finding that the number of antihypertensives did not associate with adherence is compatible with an Indian study performed in a tertiary care center [29]. They, similar to what we found here, reported that those who had experienced symptoms of HTN were significantly more likely to have low adherence to antihypertensives. In our opinion, this is a mutual relationship that patients who do not take their medicines regularly are more likely to experience hypertensive crisis and need for admission to get inpatient treatment. The same patients who were interviewed at a later date were still found to be nonadherent. It could be inferred that, despite experiencing hypertensive crisis, the patients' attitude and practice regarding taking his/her antihypertensive is not changed and they are still considered poor adherers. Likewise, the Indian article did not find any association between number of antihypertensives taken daily and adherence. This is contradictory to what was reported by a Pakistani study where it was found that higher number of patients who were taking two drugs (82% versus 18%) or more than 3 drugs (87% versus 12%) were adherent when compared to low adherers. They used the 4-item MMAS to define adherence level [13]. Self-medication and self-prescription are two quite widespread practices in Iranian patients. Although governmental authorities have consistently warned publicly about self-medication health hazards and despite advice from experts to pass legislations to limit easy access to prescription-only medications [30], unfortunately this problem still remains a health problem in Iran. Former studies on this topic focused mainly on antibiotic and analgesic self-medication which reported high prevalence of this public health concern [31]. In a similar fashion, we observed a high number of the cases who reported self-prescription and that this sample had lower adherence level. It is important to note that adherence to medication is one of the factors that contribute to acceptable BP control. There are other factors such as patient education, correct intensification of medication, and economic issues that determine good BP control [5].

We suggest that future studies focus on antihypertensive self-medication and related behavioral, economic, and social factors. It is also prudent to compare various methods to find the most effective to raise medication adherence in Iranian hypertensive patients.

Regarding strengths and limitations we faced here, in our opinion, the fact that the data were gathered from several health centers as well as pharmacies in different cities would enable us to generalize the data in a better form to the Iranian community. Particularly, the city of Karaj which is located about 30 km from the capital, Tehran, has faced an increasing growth in its population during the last 2 decades, mainly as a result of immigration from other cities.

The limitation here to be considered is that adherence is a complex phenomenon and various factors can affect that. For instance, patient awareness and knowledge about HTN and medicines prescribed, the role of the physicians in providing information and patient education, behavioral and psychological factors, doctor-patient relationship, and the like are potential factors that can be investigated in more detail in the future studies. Another limitation is related to BP recording. Since this study was done in different centers and BP recording was based on measurement by sphygmomanometer and taken by different physicians, it is likely that there might be variations in devices and maybe some variations, though we think not very significant, differences in BP readings. Our resources and limited time did not allow us to implement more accurate devices for BP monitoring such as Holter monitor.

## 5. Conclusion

Adherence to antihypertensive(s) among Iranian patients is not satisfactory and more than half of the patients had uncontrolled BP which was in direct relationship with poor adherence to medications. The patients who were overweight/obese, who had been admitted to hospital or clinic emergency services because of HTN crisis, housewives or those who were unemployed or retired, those with lower educational level, and those getting their medication directly from pharmacies without visiting their doctors to control their BP and refill prescription in hand were more likely to be low adherers. We recommend that health care professionals who manage hypertensive patients consider these factors during their routine visit of such patients in an attempt to alert the patients for better adherence.

## Figures and Tables

**Table 1:** Comparison of demographic characteristics between low and moderate/high adherence groups among 280 Iranian hypertensive patients.

	Total (*N* = 280)	Low adherence (*N* = 139)	Moderate/high adherence (*N* = 141)	Sig.
Gender				
Male	118 (42.1%)	59 (50%)	59 (50%)	0.919
Female	162 (57.9%)	80 (49.4%)	82 (50.6%)	
Age, years				
≤50	51 (18.2%)	25 (49%)	26 (51%)	0.922
>50	229 (81.8%)	114 (49.8%)	115 (50.2%)	
BMI, kg/m^2^				
<25	76 (27.1%)	28 (36.8%)	48 (63.2%)	0.011
≥25	204 (72.9%)	111 (54.4%)	93 (45.6%)	
Education				
Lower than high school diploma/illiterate	164 (58.5%)	86 (52.4%)	78 (47.6%)	0.008
High school diploma	71 (25.4%)	40 (56.3%)	31 (43.7%)	
Academic degree	45 (16.1%)	13 (28.9%)	32 (71.7%)	
Occupation				
Market/self-employed	73 (26.1%)	43 (58.9%)	30 (41.4%)	0.001
Clerk/military	41 (14.6%)	9 (22.0%)	32 (78.0%)	
Housewife	119 (42.5%)	65 (54.6%)	54 (45.4%)	
Retired/unemployed	47 (16.8%)	22 (46.8%)	25 (53.2%)	
Current smoker	49 (17.5%)	29 (59.2%)	20 (40.8%)	0.141
Insurance coverage	246 (87.8%)	119 (48.4%)	127 (51.6%)	0.253
Residence place				
Urban	273 (97.5%)	133 (48.7%)	140 (51.3%)	0.053
Rural	7 (2.5%)	6 (85.7%)	1 (14.3%)	

Sig. = significance level.

**Table 2:** Comparison of blood pressure variables between low and moderate/high adherence groups among 280 Iranian hypertensive patients.

	Total (*N* = 280)	Low adherers (*N* = 139)	Moderate/high adherers (*N* = 141)	Sig.
HTN duration, mean (±SD), year	7.23 (±5.97)	7.06 (±5.71)	7.40 (±6.23)	0.966
History of HTN crisis	99 (35.3%)	62 (62.5%)	37 (37.4%)	0.001
BP measurement by a health care provider in the last 6 months	230 (82.1%)	107 (46.5%)	123 (53.5%)	0.025
Self-awareness of BP	161 (57.5%)	81 (50.3%)	80 (49.7%)	0.795
Mean systolic BP, mmHg	136.7 (±16.2)	139.6 (±15.2)	133.8 (±16.8)	0.003
Mean diastolic BP, mmHg	83.9 (±9.0)	85.7 (±8.3)	82.1 (±9.2)	<0.001
Controlled BP in nondiabetics	120	49 (40.8%)	71 (59.2%)	<0.001
Controlled BP in diabetics	2	0	2 (100%)	0.504

**Table 3:** Comparison of number of antihypertensives and comorbidities between low and moderate/high adherence groups among 280 Iranian hypertensive patients.

	Total (*N* = 280)	Low adherers (*N* = 139)	Moderate/high adherers (*N* = 141)	Sig.
Antihypertensive therapy				
Monotherapy	127 (45.3%)	68 (53.5%)	59 (46.5%)	0.234
Combination therapy	153 (54.6%)	71 (46.4%)	82 (53.6%)	
Number of antihypertensive(s) classes taken				
1	127 (45.3%)	68 (53.5%)	59 (46.5%)	0.079
2	95 (34%)	37 (38.9%)	58 (61.1%)	
3–5	58 (20.7%)	34 (58.6%)	24 (41.4%)	
Presence of comorbidity	160 (57.1%)	77 (48.1%)	83 (51.9%)	0.629
Mean (±SD) number of comorbidities, *N* = 160 patients with at least one comorbidity	1.54 (±0.76)	1.53 (±0.75)	1.54 (±0.77)	0.984
Taking anti-hypertensive from pharmacy without doctor visit	212 (75.7%)	113 (53.3%)	99 (46.7%)	0.031

**Table 4:** Linear regression analysis of variables associated with the MMAS-8 score.

	*B*	Standard error of *B*	Significance
History of HTN crisis	−0.796	0.228	0.001
Taking antihypertensive from pharmacy without doctor visit	−0.515	0.251	0.041
BMI ≥ 25 kg/m^2^	−0.523	0.237	0.028
Constant	9.764	0.913	0.001

BMI = body mass index; HTN = hypertension.

## Additional Points

The use of the ©MMAS is protected by US copyright laws. Permission for use is required. A license agreement is available from Professor Donald E. Morisky, Sc.D., Sc.M., MSPH, Department of Community Health Sciences, UCLA Fielding School of Public Health, 650 Charles E. Young Drive South, Los Angeles, CA 90095-1772; dmorisky@ucla.edu.
